# Predicting the Structural Effects of CUG Codon Translation on Uncharacterized Proteins in *Candida albicans*

**DOI:** 10.3390/jof11090638

**Published:** 2025-08-29

**Authors:** Michaela Čermáková, Olga Heidingsfeld

**Affiliations:** 1 Department of Biochemistry, Faculty of Science, Charles University in Prague, Hlavova 2030, 128 43 Prague, Czech Republic; michaela.cermakova@natur.cuni.cz; 2 Institute of Organic Chemistry and Biochemistry of the Czech Academy of Sciences, Flemingovo náměstí 2, 166 10 Prague, Czech Republic

**Keywords:** *Candida albicans*, codon usage, CUG codon, essential gene, orphan gene, AlphaFold2

## Abstract

In the standard genetic code, the CUG triplet is translated as leucine. The pathogenic yeast *Candida albicans* and other CTG-clade yeasts contain tRNA_CAG_, which is recognized by both leucine- and serine-tRNA synthetases. The CUG codon in these yeasts is translated most often as serine, and only in 3–5% of cases as leucine. Therefore, CTG *Candida* species have unstable proteomes. The effect of serine–leucine exchange on the structure and function of proteins has only been experimentally examined for a few cases. In *C. albicans*, CUG codons occur even in genes deemed to be essential. This means that serine–leucine ambiguity either does not affect the structure and function of the respective proteins, or that the presence of these amino acids at specific positions is associated with meaningful alteration of the proteins’ function. This study employed AlphaFold2 to evaluate the potential effects of serine-to-leucine exchange in 12 proteins encoded by essential genes lacking orthologs in other yeasts and human genomes. The low homology with known proteins allowed us to make only low-confidence predictions. The analyzed proteins could be grouped into subsets based on the structural outcomes. Structural changes were observed only in four proteins. The remaining eight proteins showed no significant differences between serine and leucine variants.

## 1. Introduction

The genetic code is a universal principle uniting all living organisms. It is generally regarded as stable; nevertheless, the number of observed deviations from the standard decoding rules continues to grow. The most common coding alteration identified so far is translating one or two stop codons as an amino acid [1]. This occurs in bacteria, mitochondrial genomes of metazoa, or nuclear genomes of ciliates [1,2,3,4]. The use of all three stop codons as sense codons also occurs in nature. It has been observed in the nuclear genome of a parasitic protist, *Blastocrithidia*, and in eukaryotic marine microorganisms, *Condylostoma magnum* and *Parduzcia orbis*, where the stop codons can terminate translation only with the help of an adjacent poly(A) tail [5,6,7].

Sense-to-sense codon changes are rare, especially in eukaryotic nuclear genomes. Moreover, they are challenging to identify and experimentally verify [8]. Despite this, changes in the meaning of the CUG codon have been known since the end of the 1980s. In the standard genetic code, the CUG codon is translated as leucine. But in the set of yeast species of the subphylum *Saccharomycotina*, it is decoded as serine. This alteration was first discovered in *Candida cylindracea* [9] and then found in more *Candida species*, including the prominent human pathogens *Candida albicans*, *Candida parapsilosis*, and *Candida tropicalis* [10,11]. The set of yeast species possessing this feature has been denominated as the CUG (or CTG) clade. Later on, decoding of the CUG as alanine was observed in the yeast *Pachysolen tannophilus*, known for its ability to produce ethanol from xylose [12,13,14]. Then, Krassowski et al. reported on an additional CUG-Ser clade within the *Saccharomycotina* phylogenetic tree [13].

The key elements ensuring translation accuracy and stability are tRNA molecules. In *C. albicans*, tRNA_CAG_ has structural determinants that can be recognized by both seryl- and leucyl-tRNA synthetases. In 93–95% of cases, tRNA_CAG_ is charged with serine, while in the remaining cases, it is charged with leucine [4,15]. The dual meaning of the CUG codon in *C. albicans* is yet another intriguing alteration to the standard genetic code, challenging the traditional view that each codon corresponds to a single amino acid. Evolutionarily, the dual meaning of the CUG codon affected its usage. Potentially adverse effects resulting from an unstable proteome can be avoided by eliminating the altered codon from protein-coding sequences [16]. Indeed, a comparative genomics analysis of *C. albicans*, *Saccharomyces cerevisiae*, and *Schizosaccharomyces pombe* revealed that CUG codon ambiguity led to the disappearance of approximately 26,000–30,000 ancestral CUG codons in *Candida* spp. Only 2% of *C. albicans* CUG codons correspond to the leucine codons in *S. cerevisiae* and *S. pombe*. However, there is one more twist: over the last 272 ± 25 million years, new Ser-Leu-CUG codons have emerged, accounting for approximately 17,000 extant CUG codons in *Candida*. These codons align with those for serine- or conserved-serine-related amino acids in related yeast species [17,18]. The *C. albicans* proteome must have experienced periods of considerable instability over geological time and remains unstable to this day [15,17].

Can such instability be advantageous? One approach to addressing this question involved transforming *S. cerevisiae* with *C. albicans* Ser-tRNA_CAG_, leading to the translation of *Saccharomyces* CUG codons as both leucine and serine. The resulting *S. cerevisiae* exhibited greater tolerance to a range of stress conditions. This suggests that the synthesis of aberrant proteins due to CUG’s dual meaning induced the expression of molecular chaperones, which then protected the cells against various stresses. Indeed, Hsp104 and Hsp70 expression was increased in *S. cerevisiae* with CUG codon ambiguity [19,20]. Conversely, *C. albicans* with increased incorporation of leucine at CUG sites displayed more variable morphology and tolerance to higher levels of azole antifungals than the parental strain [21]. *C. albicans* is known for its ability to withstand stress conditions, a trait considered important for its pathogenicity [22]. The polysemous CUG codon may, therefore, be one of the factors contributing to *Candida’s* significance as a public health threat.

*C. albicans* is the leading cause of yeast infections [23]. It can coexist with healthy hosts as a harmless commensal, but when the constraints imposed by the host’s immune system or competing microflora are removed, it can cause disease, termed candidiasis [24]. Individuals at risk of *Candida* infections include patients who have undergone organ transplantation, chemotherapy, or other medical treatments that weaken the immune system. Long-term use of broad-spectrum antibiotics, which reduce prokaryotic microflora, is another risk factor that facilitates the development of candidiasis [25]. The disease can be relatively mild, with superficial and mucosal manifestations, but in deeply immunocompromised patients, the yeast can enter the bloodstream and spread to internal organs, causing life-threatening conditions [26]. Since *C. albicans* can be part of the human microbiome, candidiasis can have an endogenous origin. However, it can also be acquired externally, with nosocomial transmission being a particular concern.

Several classes of drugs have been developed to treat candidiasis. They include polyenes, azoles, echinocandins, allylamines, and pyrimidine analogs, with azoles being the most frequently used [26]. While these treatments are often effective for superficial *Candida* infections, deep-seated candidiasis is often difficult to cure. Another challenge is that the widespread use of antifungal agents exerts selective pressure, enabling the rapid evolution of *C. albicans* strains with reduced drug susceptibility. This drives the search for novel drug targets and the development of new antifungal therapies [27].

To identify potential drug targets, it is crucial to determine which molecules are essential for *C. albicans* to survive and proliferate but are absent in humans. The genome of *C. albicans* has been known and annotated for a long time, allowing for the identification of essential genes at the whole-genome level. This search has been conducted using various approaches. One of the latest studies employed transposon mutagenesis in a haploid *C. albicans* isolate [27]. Among the genes identified as essential in this study, there is a subset that lacks orthologs not only in humans but also in several other yeast species. These genes are mostly uncharacterized, and their lack of orthologs suggests that they may be considered orphan genes, which are relatively young from an evolutionary perspective [28]. Surprisingly, some of these genes contain one or more CUG codons, indicating that *C. albicans* can tolerate the dual-meaning codon even in essential genes.

We used AlphaFold2 predictions to examine how serine-to-leucine changes affect the structure of proteins encoded by these essential genes lacking orthologs. Since AlphaFold2 relies on known homologous protein structures for its predictions, the analysis of this particular protein set is less reliable and should be interpreted with caution. Nevertheless, we believe it is valuable to highlight these uncharacterized and potentially interesting proteins in an important human pathogen and to discuss structural changes in these proteins, despite some uncertainty.

## 2. Materials and Methods

The gene sequences were obtained from the *Candida* Genome Database (www.candidagenome.org) and translated into amino acid sequences using the Translate Tool available at the ExPASy Bioinformatics Resource Portal (https://web.expasy.org/translate/, accessed on 4 November 2024). Positions of Ser/Leu residues encoded by the CUG codons were found manually. Intrinsic-disorder prediction was performed using NetSurfP-2.0 [29]. The structural prediction of the uncharacterized proteins and their mutant variants was carried out using a combination of computational tools. The wild-type (WT) protein structure was retrieved from the AlphaFold Protein Structure Database, which offers highly accurate structural models based on deep learning algorithms [30]. To assess the potential structural effects of specific point mutations, selected serine residues in the WT sequence were substituted with leucine residues at positions corresponding to CTG (CUG) codons. These targeted mutations were introduced using a custom Python 3 script executed in the Google Colab environment, enabling rapid in silico modification and prediction of the mutant sequence [31]. Following model generation, the three-dimensional structures of both the WT and mutant proteins were visualized and analyzed using UCSF ChimeraX (v. 1.10) [32]. Structural alignment and comparison were conducted to identify conformational changes, potential disruption of secondary structure elements, or alterations in predicted functional regions caused by the serine-to-leucine substitutions.

## 3. Results

The selection of the open reading frames included in this study stems from the work of Segal et al. (2018) [27], who identified 1610 *C. albicans* genes essential for the growth of a haploid *C. albicans* strain under standard laboratory conditions. Among them, 17 genes were identified as having no detectable orthologs in the human genome, no orthologs in the two model yeasts *Saccharomyces cerevisiae* and *Schizosaccharomyces pombe*, and no orthologs in three major fungal pathogens *Aspergillus fumigatus*, *Cryptococcus neoformans*, and *Histoplasma capsulatum*. Twelve of these open reading frames contain at least one CUG codon and became the subject of this study (Table 1).

Due to limited homology to known protein sequences, AlphaFold2 predicts the structures of these proteins with low confidence. With this limitation in mind, we categorized them according to the predicted influence that an ambiguous CUG codon reading may have on the resulting proteins.

### 3.1. Proteins with Insignificant Ser-Leu Exchange Effect

**Figure 1:**
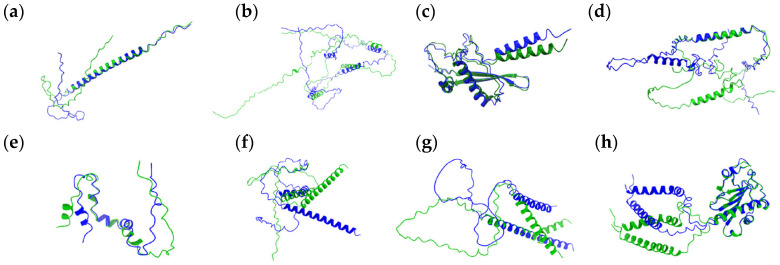
Structural alignment of proteins with no statistically significant changes in their structure. Superimposed variants are shown in green and blue. Residue identities are not labeled due to a lack of statistically relevant substitutions. Panels correspond to the following proteins: (**a**) Q5AB58, (**b**) Q5A299, (**c**) Q5A3K8, (**d**) A0A1D8PG02, (**e**) A0A1D8PF20 (TLO4), (**f**) Q5ALW9, (**g**) A0A1D8PLD5, and (**h**) A0A1D8PPD0. Green (WT—predicted structure, AlphaFold2) and blue (predicted structure with S ↔ L substitution, CoLab) indicate two protein variants aligned for comparison.

AlphaFold2 predictions suggested that in eight out of twelve ORFs, the Ser-to-Leu substitution would not significantly affect the overall protein structure (Figure 1). This finding was further supported by NetSurfP-2.0 analysis, which predicts secondary structure elements and regions of disorder. However, in several cases, Ser-Leu exchange may lead to a rearrangement in the relative positions of α-helices.

The gene C1_00020C_A has been identified as haploinsufficient for cell size, with its heterozygous deletion leading to smaller cells [33]. Although transcription of C1_00020C_A has been confirmed [34], the corresponding gene product has not yet been experimentally validated. The predicted protein (Q5AB58) is 103 amino acids long and contains three Ser-Leu exchange sites, one within a predicted helical region and two within the C-terminal unstructured region. According to NetSurfP-2.0, when leucine occupies positions 92 and 97, the segment spanning residues 89–98 may adopt a helical conformation. In contrast, AlphaFold2 predicts that the C-terminal region remains disordered regardless of the Ser-Leu substitutions (Figure 1a).

Although the gene C1_05250W_A has orthologs in *Candida lusitaniae* and *Candida guilliermondii*, it remains uncharacterized. It is predicted to encode a protein of 204 amino acids (Q5A299), 4 of which are encoded by the CUG codon. These residues are at positions 71, 100, 123, and 180, located in loops or unstructured regions of the protein (Figure 1b). While they may influence the relative positioning of the three predicted helices, their overall impact on the protein’s architecture is likely to be minimal.

The protein Q5A3K8, a potential product of the C1_12040W_A locus, is a 143-amino-acid uncharacterized protein with a conserved ortholog in *Candida dubliniensis*. Two Ser-Leu exchange sites are present at positions 94 and 101. AlphaFold modeling (Figure 1c) indicates that residue 94 lies in a surface-exposed loop, while residue 101 is located at the C-terminal end of a short α-helix spanning residues 99–101. The Ser-Leu substitution is predicted to have no significant effect on the protein’s overall structure.

The locus C1_14580C_A is annotated in the *Candida* Genome Database as an uncharacterized gene associated with a transposable element. The corresponding protein sequence matches the nucleocapsid protein of the Zorro2 retrotransposon, a member of the Zorro family of non-LTR retrotransposons in *Candida albicans* that are known to be transcriptionally active and capable of transposition [35]. The protein (A0A1D8PG02) is composed of 175 amino acids and contains a single Ser-Leu exchange site at position 10, which is located on a loop. According to AlphaFold2 (Figure 1d), this substitution is not expected to affect the overall protein structure.

*TLO4* (telomere-linked open reading frame) is one of the telomere-proximal genes of unknown function. The corresponding protein (A0A1D8PFZ0) listed in the *Candida* Genome Database consists of 66 amino acids, with the Ser-Leu exchange sites at positions 24 and 35. While transcription of the *TLO4* gene was found to be induced during oral candidiasis and regulated by Hap43p transcription factor, the existence of the Tlo4p protein has not been experimentally verified. According to the AlphaFold2 model (Figure 1e), residues 24 and 35 are both on the helix in the central part of the protein, and Ser-Leu exchange would not cause structural changes.

C2_01500W_A is an uncharacterized open reading frame predicted to encode a 144-amino-acid protein (Q5ALW9). To date, no studies have reported on this gene. A potential Ser-Leu substitution at position 102 may slightly affect the positioning of the C-terminal helix (Figure 1f). However, the overall protein structure is likely to remain unchanged.

The gene C4_01710C_A encodes a 142-amino-acid protein (A0A1D8PLD5) that has been identified as part of the transcriptional network regulating biofilm formation in *C. albicans* [36]. Among the proteins analyzed in this study, it contains the highest number of residues encoded by the ambiguous CUG codon, with seven potential Ser-Leu exchange sites. Notably, this includes a continuous stretch of five residues (positions 124–128), each of which can be either serine or leucine. Both AlphaFold2 and NetSurfP-2.0 predict this stretch to be located between the two helices in the C-terminal part of the protein, with no significant effect on the protein structure. Residues 18 and 132 are predicted by AlphaFold2 to be part of short N- and C-terminal α-helices, respectively. However, NetSurfP-2.0 classifies the N-terminal region as disordered, without a defined helical structure. In either case, Ser-Leu exchanges near the termini are also predicted to have minimal structural consequences (Figure 1g). The amino acid composition of this protein is unusual, with asparagine accounting for nearly 30% of the residues and glutamine for an additional 9%. Reflecting this low sequence complexity, the central region of the protein is predicted to be intrinsically disordered by both AlphaFold2 and NetSurfP-2.0. Such an N/Q-rich region may be capable of switching between a prion-like state and a more defined fold upon binding to an interaction partner. Although the Ser-Leu exchange sites are not located within this unstructured region, CUG codon translation may still influence overall structural alterations if the disordered segment adopts specific folds.

Although the gene C6_00270W_A remains uncharacterized, it has conserved orthologs in *Candida dubliniensis* and *Candida tropicalis*. It encodes a relatively long protein of 230 amino acids (A0A1D8PPD0), making it the longest protein analyzed in this study. AlphaFold2 predictions suggest that the protein is relatively well structured. Two potential Ser-Leu exchange sites are present at positions 100 and 107, both located within a surface-exposed loop, and are not expected to affect the overall protein structure (Figure 1h).

### 3.2. Proteins with Predicted Serine Phosphorylation

Leucine-serine exchange can have more significant consequences if the serine is phosphorylated. Sárkány et al. suggested that some CUG-encoded serines, particularly within signal transduction proteins, could be post-translationally modified [37]. However, to the best of our knowledge, there are no confirmed examples of a CUG-encoded serine in *Candida albicans* that have been shown to be directly phosphorylated in vivo or in vitro. Despite this, we used Prosite to find serine residues in our sequences encoded by CUG codons, which might be phosphorylated. Potential phosphorylation sites were identified in five proteins, one site in each of them: Q5AB58, Q5A3K8, A0A1D8PG02, A0A1D8PFZ0, and A0A1D8PLD5. The usage of these sites is questionable; however, at least in the case of A0A1D8PG02, the nucleocapsid protein of the Zorro-2 retrotransposon, serine phosphorylation would not be surprising.

### 3.3. Proteins Affected by Ser-Leu Exchange

**Figure 2:**
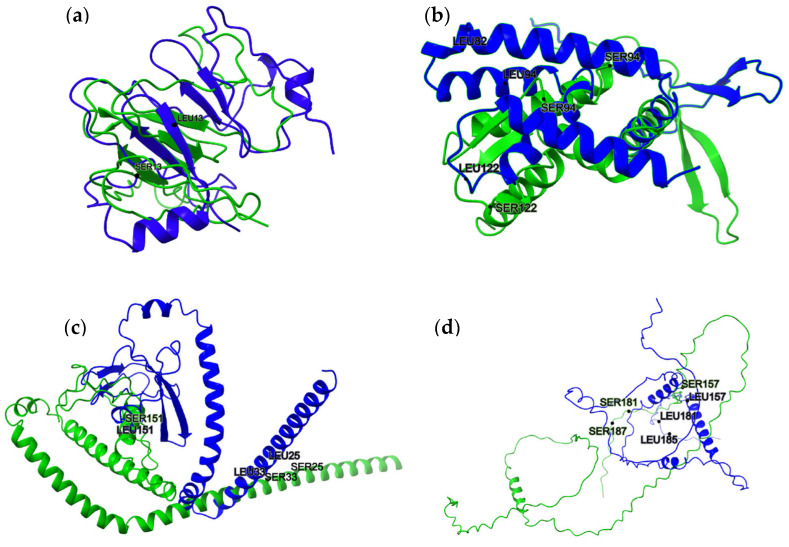
Structural alignment of proteins with statistically significant differences at the indicated residue positions. Superimposed structures highlight substitutions between serine (SER) and leucine (LEU) residues in positions (**a**) Q59KZ4 (SER13 ↔ LEU13), (**b**) Q5ACW4 (SER82 ↔ LEU82, SER94 ↔ LEU94 and SER122 ↔ LEU122), (**c**) Q5A7K2 (SER25 ↔ LEU25, SER33 ↔ LEU33 and SER151 ↔ LEU151), and (**d**) A0A1D8PNC2 (SER157 ↔ LEU157, SER181 ↔ LEU181 and SER185 ↔ LEU185). Green (WT—predicted structure, Alphafold2) and blue (predicted structure with Ser ↔ Leu substitution, CoLab) indicate two protein variants aligned for comparison. Labels indicate residue identity and position.

In four proteins, AlphaFold2 prediction indicated that Ser-Leu exchange may induce more profound changes. C1_04440W_A is an uncharacterized ORF with no references in the literature, to the best of our knowledge. It is predicted to encode a 122-amino-acid protein (Q59KZ4) with a Ser-Leu site at position 13. The AlphaFold2 models show notable differences: the Ser13 variant appears partially disordered and includes β-sheets, while the Leu13 variant adopts a more helical conformation (Figure 2a). Of note, the NetSurfP-2.0 prediction suggests that both variants are structurally similar, containing β-sheets and disordered regions.

C2_00310W_A encodes a 148-amino-acid protein (Q5ACW4) that contains three Ser-Leu exchange sites. This protein remains uncharacterized, with no reports in the literature to date. According to AlphaFold2 predictions (Figure 2b), both the serine- and leucine-containing variants are relatively well-structured, featuring a central helical core along with some β-structures. While the overall fold is largely preserved between the two versions, local topological differences are evident. The variant containing serines at all three CUG-encoded positions appears more compact. In contrast, the leucine-containing version displays extended β-strands and longer, more disordered loops. NetSurfP-2.0 also predicts the serine variant to be more compact; however, it does not identify any β-structures in either version of the protein.

C3_00120W_A encodes an uncharacterized protein consisting of 208 amino acids (Q5A7K2) and contains three Ser-Leu exchange sites. Both the serine- and leucine-containing variants are characterized by a prominent extended α-helix. In the serine-containing version, this helix is bent, resulting in a more compact overall structure. While both variants include β-structures, the serine version appears to have more tightly packed strands. Likewise, the loop regions in the serine-containing variant are more compact, exhibiting tighter turns and shorter loops compared to the leucine variant (Figure 2c).

The final protein in the analyzed panel, A0A1D8PNC2 (192 amino acids), encoded by the C5_02130W_A locus, is uncharacterized and contains three Ser-Leu exchange sites. Both the serine- and leucine-containing variants feature a prominent helical structure along with a substantial proportion of disordered regions. In this case, the leucine-containing variant appears more structured and compact compared to the more extended and disordered serine-containing version (Figure 2d).

**Figure 3:**
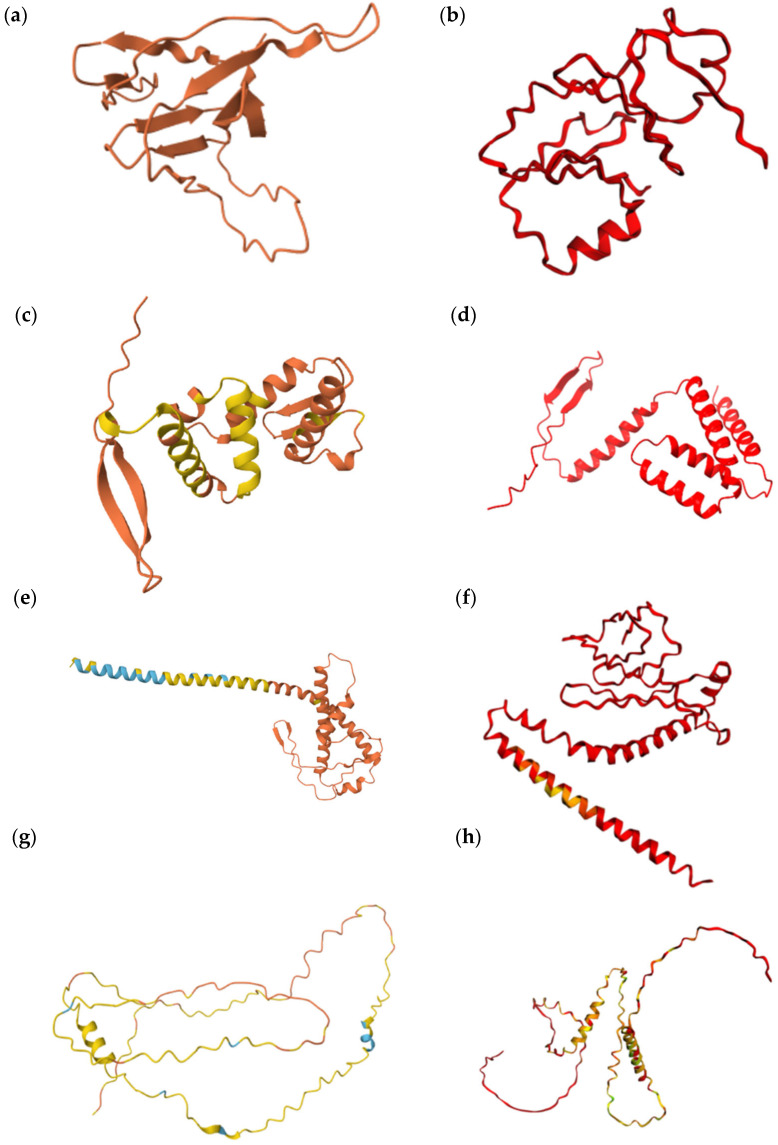
Predicted protein structures. Wild-type proteins were predicted with AlphaFold2, whereas S–L exchange variants were modeled using Colab: (**a**) Q59KZ4 WT, (**b**) Q59KZ4 (Ser13 ↔ Leu13), (**c**) Q5ACW4 WT, (**d**) Q5ACW4 (Ser82 ↔ Leu82, Ser94 ↔ Leu94, and Ser122 ↔ Leu122), (**e**) Q5A7K2 WT, (**f**) Q5A7K2 (Ser25 ↔ Leu25, Ser33 ↔ Leu33, and Ser151 ↔ Leu151), (**g**) A0A1D8PNC2 WT, (**h**) A0A1D8PNC2 (Ser157 ↔ Leu157, Ser181 ↔ Leu181, and Ser185 ↔ Leu185). Model confidence levels differed between AlphaFold2 and Colab predictions. Model Confidence AlphaFold2: very high (pLDDT > 90, royal blue), high (pLDDT > 70; cyan), low (pLDDT > 50, yellow), and very low (pLDDT < 50, orange); Model Confidence CoLab: very high (pLDDT > 90, royal blue), confident pLDDT ~80, cyan), OK (pLDDT ~70, green), low (pLDDT ~60, yellow), and very low (pLDDT < 50, red).

Figure 3 illustrates the confidence levels of the protein models in which the Ser-Leu exchange is predicted to cause structural changes.

## 4. Discussion

The existence of essential orphan genes in *C. albicans* that retain CUG codons highlights a paradox at the intersection of genetic code evolution, protein stability, and species-specific adaptation. Genes lacking homologs in other organisms are often considered to be evolutionarily young. This raises the following question: How could *C. albicans* have entrusted essential functions to genes that are both taxonomically unique and potentially unstable due to the dual meaning of the CUG codon?

One possibility is that the impact of the CUG codon is negligible in these proteins due to their structural robustness, particularly if the Ser-Leu substitutions occur in disordered or functionally tolerant regions. Alternatively, the dual meaning of CUG may confer a functional advantage, introducing a subtle form of adaptive flexibility that allows proteins to respond to changing conditions. Indeed, both scenarios have been documented in the literature. For example, the *C. albicans* translation initiation factor 4E contains a CUG site where decoding as serine or leucine results in altered protein properties, supporting the idea that codon ambiguity plays a regulatory role [38]. Conversely, the exo-β-1,3-glucanase from *C. albicans* showed no detectable structural differences upon Ser-Leu substitution [39], nor did the secreted aspartic protease from *C. parapsilosis*, another CTG-clade species [40]. These contrasting outcomes reinforce the view that the structural and functional consequences of CUG codon usage are context-dependent.

However, it remains unclear whether these codons have persisted in the essential orphan genes analyzed in this study, simply because these genes are evolutionarily young and have not yet undergone purging, or whether the CUGs were introduced after the genes became essential. The latter scenario, de novo insertion of ambiguous codons into already essential genes, seems less likely. Nonetheless, the retention of CUG codons in these genes suggests that their presence is at least selectively neutral, if not advantageous.

To address whether the presence of CUG codons in these essential orphan genes reflects protein robustness or adds a potential functional advantage, we employed AlphaFold2 and other structural prediction tools to compare the serine- and leucine-containing variants of selected proteins. These analyses aimed to distinguish between cases where the Ser-Leu substitution has minimal structural impact—suggesting robustness—and those where the exchange may induce conformational differences that could confer functional versatility. However, it is important to note that all predictions must be interpreted with caution. Due to the lack of sequence homology and structural templates for orphan proteins, the confidence of these models is inherently limited, particularly in disordered regions or unconventional folds. It is also important to emphasize that most of these proteins have not been experimentally confirmed at the protein level; their existence remains predicted based on gene models and computational annotation.

In eight cases, AlphaFold2 modeling suggested that the Ser-Leu exchange would not lead to significant structural changes. These proteins typically contained extensive unstructured regions, which may contribute to their apparent tolerance to amino acid variation at the CUG-encoded sites. The flexibility of intrinsically disordered regions likely buffers the structural impact of Ser-Leu substitution, supporting the idea of protein robustness. On the other hand, unstructured regions have the potential to adopt defined conformations upon interaction with other proteins, nucleic acids, or other types of ligands. An open question is whether the translation of the CUG codon can contribute to such structural changes. In this study, this issue is particularly relevant for proteins Q5AB58, A0A1D8PLD5, and A0A1D8PNC2, which contain large disordered regions with Ser-Leu exchange sites located either within these regions or in their proximity.

In four of the analyzed proteins, AlphaFold2 modeling predicted structural alterations resulting from Ser-Leu exchange at CUG-encoded positions. The case of Q59KZ4 is particularly striking. It contains only a single CUG site, yet the predicted structural difference between the serine and leucine variants was substantial. This observation is difficult to interpret, as it would imply that a single amino acid substitution exerts a disproportionately large effect on the overall fold. This outcome, while possible in theory, remains speculative and without experimental validation. Two other proteins, Q5ACW4 and Q5A7K2, each harbor three CUG sites. In both cases, the serine-containing variants appeared more compact compared to their leucine counterparts. This shift in conformation may point to differences in protein flexibility or stability and raises the possibility that the dual encoding could confer functional or regulatory adaptability under varying cellular conditions. A similar observation was made for the last protein, A0A1D8PNC2, which also contains three CUG sites. In this case, however, the leucine-containing variant appeared more structured and compact than the serine variant. This inverse pattern further supports the notion that Ser-Leu exchange can influence protein conformation in a context-dependent manner, potentially contributing to functional diversity.

Another layer of complexity arises from the potential post-translational modification of CUG-encoded serines through phosphorylation. While phosphorylation of serines resulting from CUG translation has not yet been experimentally confirmed in *C. albicans*, it remains a plausible regulatory mechanism—particularly in proteins exposed to dynamic cellular processes. One notable candidate is the predicted Zorro-2 nucleocapsid protein A0A1D8PG02, where the CUG-encoded serine is located in a region likely to be accessible and conformationally flexible. Given the role of nucleocapsid proteins in retrotransposon packaging and regulation, phosphorylation at this site could modulate protein–protein or protein–RNA interactions, influence assembly dynamics, or serve as a regulatory switch in retrotransposition [41].

In this study, we aimed to draw attention to a subset of potentially interesting and under-studied genes, essential orphan genes in *C. albicans* that retain CUG codons. These genes occupy a unique intersection of evolutionary novelty, genetic code ambiguity, and functional indispensability. They have not been studied from the perspective of virulence, but their essentiality makes them relevant as potential drug targets. While our structural predictions provide initial insights, a central question remains unresolved: How can *C. albicans* rely on proteins encoded by genes that are both unique and subject to the dual interpretation of one of the codons?

## Figures and Tables

**Table 1:** List of the genes included in this study. Systematic gene names are according to the *Candida* Genome Database, and protein accession numbers are according to Uniprot.

Systematic Name	Uniprot Accession	Protein Length (AA)	Number of CUG Codons	Positions of the CUG-Encoded AA	Predicted Ser/Leu Exchange Effect
C1_00020C_A	Q5AB58	103	3	24, 92, 97	P, insignificant
C1_04440W_A	Q59KZ4	122	1	13	structure altered
C1_05250W_A	Q5A299	204	4	71, 100, 123, 180	insignificant
C1_12040W_A	Q5A3K8	143	2	94, 101	P, insignificant
C1_14580C_A	A0A1D8PG02	175	1	10	P, insignificant
C1_14590C_A (*TLO4*)	A0A8H6F4J0	66	2	24, 35	P, insignificant
C2_00310W_A	Q5ACW4	148	3	82, 94, 122	structure altered
C2_01500W_A	Q5ALW9	144	1	102	insignificant
C3_00120W_A	Q5A7K2	208	3	25, 33,151	structure altered
C4_01710C_A	A0A1D8PLD5	142	7	18, 124, 125, 126, 127, 128, 132	P, insignificant
C5_02130W_A	A0A1D8PNC2	192	3	157, 181, 185	structure altered
C6_00270W_A	A0A1D8PPD0	230	2	100, 107	insignificant

AA—amino acids; P—phosphorylation.

## Data Availability

The data presented in this study are available in Laboratory of Pathogenic Yeasts at https://natur.cuni.cz/chemie/katedry-a-pracoviste/katedra-biochemie/veda-a-vyzkum/laborator-biochem accessed on 4 November 2024.

## Abbreviations

The following abbreviations are used in this manuscript:

AA	Amino Acid
ORF	Open Reading Frame
P	Phosphorylation
